# IL-6 Promotes the Proliferation and Immunosuppressive Function of Myeloid-Derived Suppressor Cells via the MAPK Signaling Pathway in Bladder Cancer

**DOI:** 10.1155/2021/5535578

**Published:** 2021-04-23

**Authors:** Zhong Zheng, Xinyi Zheng, Yiwen Zhu, Zhixian Yao, Weiguang Zhao, Youjia Zhu, Feng Sun, Xingyu Mu, Yong Wang, Wanqing He, Zhihong Liu, Ke Wu, Junhua Zheng

**Affiliations:** ^1^Department of Urology, Shanghai General Hospital, School of Medicine, Shanghai Jiao Tong University, Shanghai, China; ^2^Department of Pharmacy, Huashan Hospital, Fudan University, Shanghai, China; ^3^School of Rehabilitation Science, Shanghai University of Traditional Chinese Medicine, Shanghai, China; ^4^The Second School of Medicine, Wenzhou Medical University, Wenzhou, China; ^5^Department of Urology, Shanghai Jiangqiao Hospital, Jiading Branch, Shanghai General Hospital, Shanghai, China; ^6^Student Innovation Center, Shanghai Jiao Tong University, Shanghai, China

## Abstract

Muscle-invasive bladder cancer (MIBC) is characterized by a highly complex immune environment, which is not well understood. Interleukin-6 (IL-6) is generated and secreted by multifarious types of cells, including tumor cells. This study was aimed at demonstrating that the levels of IL-6 and the number of myeloid-derived suppressor cells (MDSCs), with a positive correlation between them, increased in MIBC tissues, promoting MIBC cell proliferation, especially in patients with recurrence. In coculture analysis, MDSCs, with the stimulation of IL-6, could significantly lower the proliferation ability of CD4^+^ or CD8^+^ T lymphocytes. Further, this study demonstrated that IL-6 could upregulate the mitogen-activated protein kinase (MAPK) signaling pathway in MDSCs. The MAPK signaling inhibitor, aloesin, partially reversed the effects of IL-6 on MDSCs. These data suggested that IL-6 promoted MIBC progression by not only accelerating proliferation but also improving the immune suppression ability of MDSCs through activating the MAPK signaling pathway.

## 1. Introduction

Bladder cancer (BC) has the 14th and 9th highest rates of mortality and incidence among all types of cancers [1], accounting for approximately 900,000 newly diagnosed cases each year, with 250,000 deaths occurring during the same time span [2]. Nearly 75% of patients are diagnosed with non-muscle-invasive BC (NMIBC) and close to 25% with muscle-invasive BC (MIBC) [3]. Similar to other solid tumors, MIBC is classified as immunogenic cancer, with numerous tumor-infiltrating lymphocytes (TILs) in the tumor microenvironment (TME) [4]. Besides tumor cells, the TME includes fibroblasts, endothelial cells, mast cells, macrophagocytes (M*φ*), TILs, and myeloid-derived suppressor cells (MDSCs) [5]. In particular, TILs contribute to the adaptive immunological response in opposition to cancer cells and exert anticarcinoma effects in most malignant tumors, including BC, colorectal cancer, lung cancer, and melanoma [6]. Nevertheless, the dysregulation of immunosuppressive TME is intensively associated with the development from NMIBC to MIBC [7]. It is, thus, possible that the immunosuppressive functions of TILs in MIBC tumors are prompted, explaining the poor outcomes in MIBC. However, the mechanisms underlying the peculiar pathophysiologic behavior of TILs in MIBC are unclear.

Heterogeneous MDSCs, a cluster of immature myeloid-derived cells, are both immunosuppressive and angiogenic. They result from and suppress inflammatory conditions that support tumor growth [8] by stimulating the accumulation of regulatory T lymphocytes and suppressing responses initiated by cytotoxic T cells [9]. In patients bearing solid tumors, including MIBC, the interleukin 6 (IL-6) levels are often elevated and inversely associated with prognosis [10]. IL-6 can be generated in a local cytokine niche and is produced by diverse tissues, notably proinflammatory cell types, containing dendritic cells (DCs), M*φ*, MDSCs, T lymphocytes, cancer-associated fibroblasts, and endothelial cells [11]. IL-6 knockdown can affect the immune status, inducing the infiltration of MDSCs. Hence, the mechanisms underlying IL-6-mediated MDSC penetration and functionality should be further explored [12].

Mitogen-activated protein kinases (MAPKs) are serine-threonine protein kinases that modulate a large number of cellular processes, such as innate immunity, survival, apoptosis, differentiation, proliferation, and inflammation [13]. In the wake of tissue damage or pathogen infection, the initiation of pattern recognition receptors in the cell plasma and on the cellular surface of innate immune cells activates a majority of MAPK subfamilies, that is, p38 MAPK, extracellular signal-regulated kinase (ERK), and Jun N-terminal kinase subfamilies. In combination with the upregulation of interferon-regulatory factor as well as nuclear factor-*κ*B, MAPK activation leads to the upregulation of a plethora of relevant genes that modulate the immune responses together [13]. The ERK signaling pathway mediates the transition of MDSCs. Its downregulation is correlated with MDSC differentiation. The dysregulation of MAPK signaling pathways promotes the pathogenesis of malignant tumors [14].

This study was aimed at exploring the mechanisms by which IL-6 promotes the immunosuppressive functions and proliferation of MDSCs via the MAPK signaling pathway in MIBC.

## 2. Materials and Methods

### 2.1. Patient Materials

Malignant lesions and adjacent normal controls were obtained from 200 patients (aged 53–88 years) diagnosed with MIBC who underwent radical cystectomy or transurethral resection of the bladder tumor (TURBT). Specimens were obtained from Shanghai General Hospital, Shanghai, China. The removal of tissues and subsequent analyses were approved by the ethics committee of the hospital and performed after obtaining informed consent from the patients' relatives.

### 2.2. *In Vivo* Experiments

C57BL/6 mice (Shanghai Model Organisms Center, China), aged 6–8 weeks, were subcutaneously (s.c.) injected with MB49 cells (2 × 10^4^) and raised under pathogen-free conditions in the Shanghai General Hospital Animal Center. In the IL-6 treatment group, the mice were randomly divided into the experimental group (*n* = 20) and control group (*n* = 20). The grouping conditions in the MDSC transfer group were identical with those in the IL-6 treatment group, but it was launched after the sacrifice of mice on the 30th day in the IL-6 control group owing to the requirement of MDSCs. The BC tissues of the mice in the IL-6 control group after relevant analysis were subsequently used for MDSC separation and transfer. All the *in vivo* experiments were launched 10 days (the average number of days for the visible tumor node) after tumor implantation. In the IL-6 treatment group, the mice were intraperitoneally injected with phosphate-buffered saline (PBS) or recombinant murine IL-6 (aa 20-357, 20 ng/g body weight; carrier-free, R&D Systems) at 10 a.m. every day. In addition, MDSCs from BC tissues (5 × 10^5^ cells/mouse) mentioned earlier were adoptively transferred to BC mice via tail vein injection every 7 days, and PBS as a vehicle was administered to the control group. The tumor growth rate was calculated by measuring the estimated tumor volume with a digital caliper every 5 days. The estimated tumor volume was determined using the canonical formula: volume (cm^3^) = 1/2 × (width)^2^ × length. After 30 days, 10 mice randomly extracted from each group were euthanized. The tumor tissues were collected for flow cytometry and MDSC separation for further experiments, including coculture analysis, quantitative reverse transcription polymerase chain reaction (qRT-PCR), and RNA sequencing, under specific conditions. The remaining 10 mice per group were kept for survival analysis. The survival curves for all groups were established using the Kaplan–Meier criteria. The mice were considered expired when the tumor volume reached 2.5 cm^3^ posttreatment in concordance with the regulations released by the Institutional Animal Care and Use Committee. The animal study was scrutinized and approved by the Institutional Review Board of Shanghai Jiao Tong University.

### 2.3. Immunohistochemistry

The slices from the formalin-fixed paraffin-embedded blocks of MIBC specimens were obtained, and an anti-IL-6 antibody (ab233706, Abcam) was applied for immunostaining to investigate the expression of IL-6. Slices (4 *μ*m) of MIBC were loaded on poly-L-lysine-coated slides with air drying and deparaffinization. Subsequently, peroxidases were blocked with 5% hydrogen peroxide in 50% methyl alcohol at 20°C for 20 min. Then, antigen retrieval was performed using a microwave oven for 20 min in citrate buffer solution. The slices were treated with a monoclonal rabbit anti-IL-6 antibody for 12 h at 4°C after blocking nonspecific binding. They were washed with PBS and treated with biotinylated goat anti-rabbit IgG (ab150077, Abcam) for 30 min, followed by rinsing with PBS. The immune reactivity was examined using the avidin-biotin-peroxidase complex method utilizing VECTASTAIN ABC Kit (PK-4001, Vector). Then, the slices were stained with hematoxylin, dehydrated, cleared, and mounted. The tissues manifesting brown staining in the membrane, nucleus, or cytoplasm were considered positive. The average optical density (AOD) was analyzed using ImageJ software (version 2.0.0, Rawak Software, Inc., Germany) to investigate the correlation between IL-6 levels and MDSCs.

### 2.4. IL-6 Enzyme-Linked Immunosorbent Assay

The IL-6 enzyme-linked immunosorbent assay (ELISA) was applied according to the product specification (ab178013 for humans and ab100713 for mice, Abcam). Duplicate aliquots of the supernatant were dispersed in the flat-bottom 96-well plates. The assay was implemented triply on independent samples.

### 2.5. Flow Cytometry and MDSC Isolation

Fresh tumor and adjacent tissues were used to prepare single-cell suspensions. The antibodies used for flow cytometry were provided by BioLegend and are listed in Table S1. The cells were examined using a FACSCalibur Flow Cytometer (Becton Dickinson). The cells were stained with carboxyfluorescein succinimidyl ester (CFSE) and analyzed using a flow cytometer (Thermo Fisher) following the manufacturer's protocol. The cells were fixed, permeabilized, and stained with anti-IL-6-FITC (501104, BioLegend) for 30 min to detect the expression of IL-6. Data were analyzed using FlowJo software (version 10.7.1, Treestar). For MDSC flow cytometric sorting, 1 × 10^7^ cells/mL single-cell suspensions made from malignant tissues derived from tumor-bearing mice were applied with conjugated antibodies shown in Table S1 for 20 min on ice in staining buffer (1% FBS in PBS). Then, the cells were rinsed with PBS, and the samples were sorted using a BD Influx. The 1.5-drop pure sort mode was selected to obtain cells with high purity. Subsequently, isolated MDSCs were maintained for experiments as follows. The cell culture passage number was no more than 10 for all isolated cells, and three replicates were performed in each passage.

### 2.6. RT-PCR Analyses of *IL6*, *Arg1*, and *iNOS*

Total RNA was obtained using the RNeasy Mini Kit (27104, Qiagen). Reverse transcription was performed using the TaKaRa RNA PCR kit (RR012A, TaKaRa) following the manufacturer's protocols. The primers were as follows: *IL6* (forward 5′-GCTTCCCTCAGGATGCTTGT-3′, reverse 5′-ATTAACTGGGGTGCCTGCTC-3′), *Arg1* (forward 5′-AGGTTAGAGGCCCAAACTGC-3′, reverse 5′-TGTCAGCTCCACCGACTTTC-3′), and *iNOS* (forward 5′-CTGAAGATACGGACGGGTCG-3′, reverse 5′-CGATGAAGTCGGGGTCGTAG-3′) for humans and *IL6* (forward 5′-GCCCTCTAGTGGTGCTTGTT-3′, reverse 5′-ACTGCAGGCCAGTTACATCC-3′), *Arg1* (forward 5′-TGCATGCAGTGAAGTGTTGC-3′, reverse 5′-AGCCCTAAACCTCCCAATGC-3′), and *iNOS* (forward 5′-CTGAAGATACGGACGGGTCG-3′, reverse 5′-CGATGAAGTCGGGGTCGTAG-3′) for mice. The SYBR Green PCR Master Mix (4309155, Thermo Fisher) and a real-time PCR machine (Applied Biosystems) were used in the reactions. All experiments were performed in triplicate.

### 2.7. Immune Suppression

Lymphocytes from plasma were isolated by the density-gradient centrifugation using Ficoll-Hypaque solution (Solarbio). CD8^+^ microbeads (130-045-201, Miltenyi Biotec) or CD4^+^ microbeads (130-045-101, Miltenyi Biotec) were used for T cell sorting. The freshly isolated CD8^+^ T cells or CD4^+^ T cells were subsequently cultured in 96-well round-bottomed plates under the conditions that a complete culture medium contained 500 ng/mL soluble anti-CD28 (T9-577-T100, American Research Products) and 500 ng/mL soluble or immobilized anti-CD3 (T1P1D4∗C6, American Research Products) for T cell stimulation and activation. The CD8^+^ or CD4^+^ T cell proliferation was evaluated using the CFSE Cell Division Tracker Kit (423801, BioLegend). MDSCs were added to the coculture at different ratios to CD8^+^ T cells or CD4^+^ T cells for 72 h (CD8^+^ T cells/CD4^+^ T cells to MDSCs = 4 : 1 and 2 : 1), 48 h after T cell stimulation. The cells were then collected and treated with the APC-CD8^+^ T cell antibody (344722 for humans and 100712 for mice, BioLegend) or APC-CD4^+^ T cells (357408 for humans and 100412 for mice, BioLegend) prior to flow cytometry. The cell culture passage number was no more than 10 for all isolated cells, and three replicates were performed in each passage. All experiments were performed in triplicate.

### 2.8. Western Blot Analysis

The cells were completely lysed in proteinase-containing radioimmunoprecipitation assay buffer inhibitor (4693132001, Complete Roche). Protein quantification was applied using the protein assay reagent (5000001, Bio-Rad). Further, 10 *μ*g of total protein was used for subsequent western blot (WB) analysis. The lysates were electrophoresed and transferred to Hybond nitrocellulose paper (FFN08, Beyotime). The nonspecific binding was blocked with 5% skimmed milk in PBS with 0.1% Tween-20 (P0220, Beyotime). For target protein examination, the blots were treated with the primary anti-human or anti-mouse antibodies at 4°C overnight. The blots were rinsed with PBST and treated with the HRP-conjugated goat anti-rabbit IgG antibody (ab6702, Abcam) for 60 min at 20°C. The target proteins were detected using the ECL WB substrate kit (ab65623, Abcam), and blots were applied to Amersham Imager 600 (GE Healthcare Life Sciences). The densitometry readings/intensity ratio of each band was analyzed by using ImageJ software (version 2.0.0, Rawak Software, Inc. Germany). All experiments were performed in triplicate. All relevant antibodies are listed in Table S2.

### 2.9. RNA Sequencing

Total RNA was obtained and enriched for mRNA using MicroPoly A Purist (AM1919, Ambion). The RNA quantity and quality were assessed using Bioanalyzer 2100 (Agilent Technologies). RNA sequencing was performed using the Illumina HiSeq 2000 platform (Illumina) for each sample. Digital gene expression profiles were used to analyze the differential gene expression between groups. The pathway enrichment was analyzed to further elucidate the cellular mechanisms.

### 2.10. Statistical Analysis

The results were expressed as means ± standard deviation. Analyses of variance and unpaired or paired-sample Student's *t*-tests were used for *in vitro* and *in vivo* experiments.

## 3. Results

### 3.1. IL-6 Levels Were Elevated in MIBC Cells and Associated with Cancer Progression

**Figure 1:**
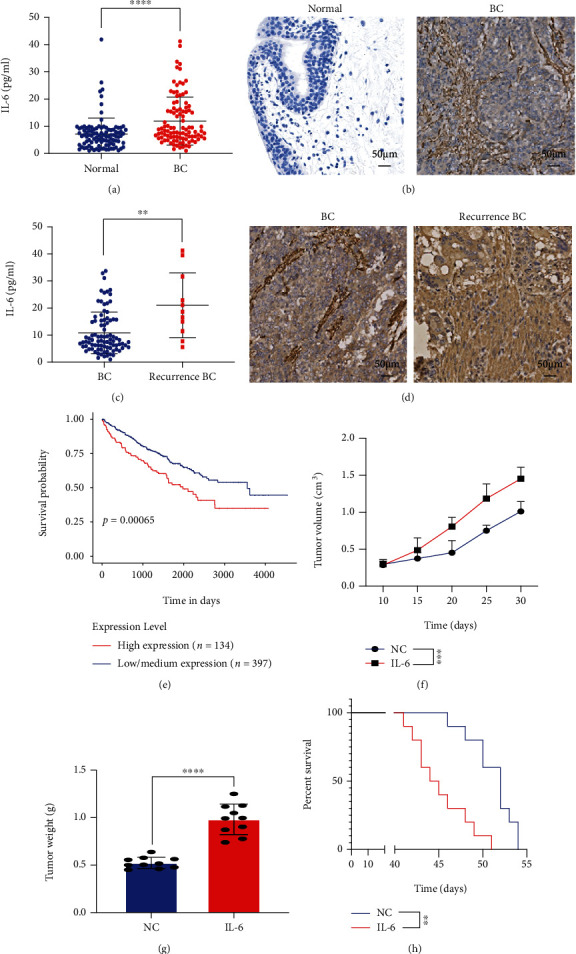
Interleukin 6 (IL-6) levels increased in muscle-invasive bladder cancer (MIBC) and promoted cancer progression. (a) IL-6 levels in MIBC tissues and normal urothelium determined by the enzyme-linked immunosorbent assay (ELISA). (b) Immunochemistry results for IL-6 in MIBC tissues and normal urothelium. (c) IL-6 levels in MIBC tissues and recurrent MIBC determined by ELISA. (d) Immunochemistry results for IL-6 in MIBC tissues and recurrent MIBC. (e) Prognosis of patients with high or low IL-6 expression according to TCGA database. (f) MIBC tumor volume with or without IL-6 treatment (*n* = 10). (g) Tumor weight with or without IL-6 treatment (*n* = 10). (h) Survival time for mice with MIBC with or without IL-6 treatment (*n* = 10). Mean ± SD, ^∗∗^*P* < 0.01, ^∗∗∗^*P* < 0.005, and ^∗∗∗∗^*P* < 0.001.

MIBC tissues (*n* = 200) were collected and compared with the normal urothelium (*n* = 200), which included 11 recurrent MIBC tissues to test the IL-6 expression level in MIBC cells. ELISA showed that the IL-6 level was higher in MIBC tissues than in adjacent noncancer tissues (Figure 1(a)). Immunochemistry showed that IL-6 staining was more obvious in MIBC tissues than in adjacent tissues (Figure 1(b)). Also, IL-6 levels were compared between recurrent and primary MIBC. ELISA showed that the IL-6 levels were higher in recurrent MIBC tissues than in primary MIBC tissues. Immunochemistry also showed that IL-6 staining was more obvious in recurrent MIBC tissues than in primary MIBC tissues (Figures 1(c) and 1(d)). According to TCGA database, high IL-6 expression was associated with a worse prognosis in patients with MIBC than in patients with low IL-6 levels (Figure 1(e)). *In vivo*, MIBC mouse models were treated with IL-6 for the analyses of tumor growth rates and tumor weights. IL-6 promoted MIBC growth (Figures 1(f)–1(g)). Mice with MIBC treated with IL-6 showed shorter survival times compared with untreated control mice (Figure 1(h)). These results showed that IL-6 levels were elevated in MIBC tissues, especially in cases of recurrence, and promoted MIBC progression by accelerating proliferation.

### 3.2. Number of MDSCs Increased in MIBC Tissues and Promoted Cancer Progression

**Figure 2:**
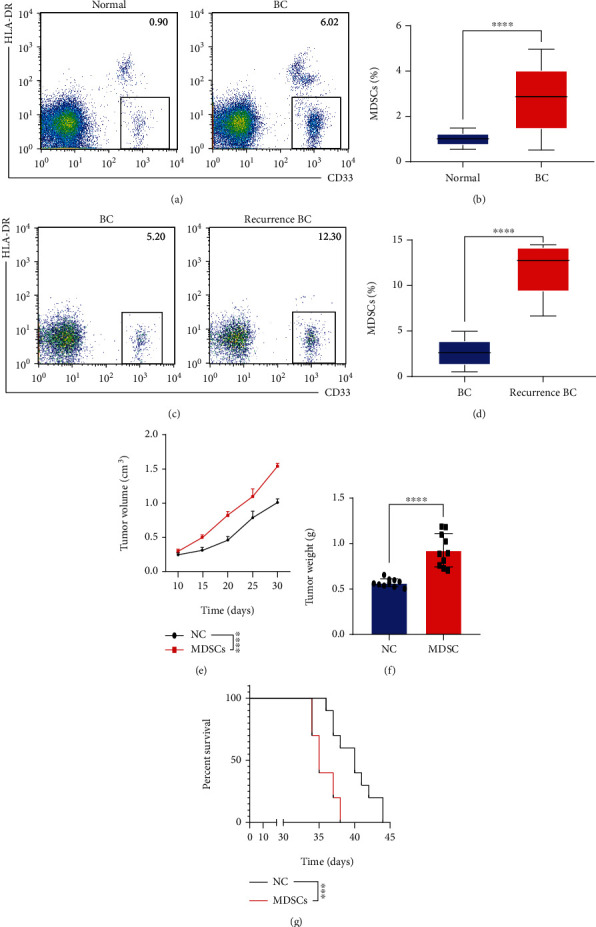
Myeloid-derived suppressor cells (MDSCs) increased in MIBC tissues and promoted cancer progression. (a, b) Flow cytometry and statistical analysis of the proportion of MDSCs in MIBC tissues and normal urothelium from 200 patients. (c, d) Flow cytometry and statistical analysis of the proportion of MDSCs in recurrent MIBC tissues and in MIBC tissues. (e) Tumor growth rates in a MIBC mouse model with or without MDSC treatment (*n* = 10). (f) Tumor weight in a MIBC mouse model with or without MDSC treatment (*n* = 10). (g) Survival time of a MIBC mouse model with or without MDSC treatment (*n* = 10). Mean ± SD, ^∗∗∗^*P* < 0.005, and ^∗∗∗∗^*P* < 0.001.

MDSCs are a heterogeneous cluster of immune cells deriving from the myeloid lineage with an immune suppression role in MIBC. MDSCs were identified as CD45^+^CD11b^+^HLA-DR^−^CD33^+^ by flow cytometry as previously studied [15], and the frequency of MDSCs was found to be higher in MIBC tissues than in the normal urothelium (Figures 2(a) and 2(b)). The increase in the number of MDSCs was greater in recurrent MIBC than in primary MIBC tissues (Figures 2(c) and 2(d)). *In vivo*, MDSCs were adoptively transferred into mice with MIBC, and cancer progression was evaluated. MDSCs increased tumor growth rates (Figure 2(e)). The tumor weights were higher in the MDSC-treated group compared with the control group (Figure 2(f)). Based on survival time, MDSC transfer in the MIBC mouse model was related to a worse prognosis (Figure 2(g)). Taken together, MDSCs increased in MIBC tissues and promoted cancer progression.

### 3.3. IL-6 Promoted MDSC Proliferation in MIBC Tissues

**Figure 3:**
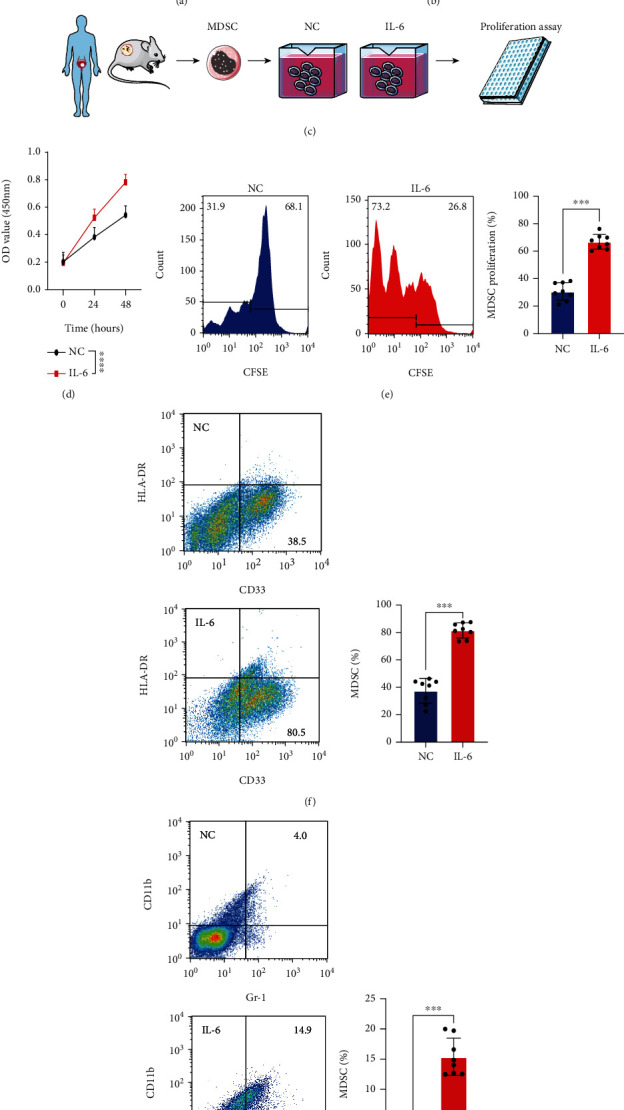
IL-6 promoted MDSC proliferation in MIBC tissues. (a, b) Correlation between IL-6 (AOD and MFI) and MDSCs in MIBC tissues. (c) Experimental design of MDSC extraction and IL-6 treatment. (d) CCK-8 assays were used to test MDSCs with or without IL-6 treatment. (e) MDSC proliferation rates were evaluated according to fluorescence attenuation. (f, g) Flow cytometry images and statistical analysis of MDSCs from humans and mice with or without IL-6 treatment. Mean ± SD, ^∗∗∗^*P* < 0.005, and ^∗∗∗∗^*P* < 0.001. AOD: average optical density; MFI: median fluorescence intensity.

IL-6 is closely related to several immune responses, including MDSC-induced immune suppression. A total of 100 MIBC tissues were collected to explore the relationship between IL-6 and MDSCs. Immunohistochemistry and flow cytometry were used to evaluate IL-6 levels, and flow cytometry was used to quantify MDSCs. The IL-6 levels were positively related to MDSCs (Figures 3(a) and 3(b)). *In vitro*, MDSCs were sorted from mouse MIBC tissues and treated with or without IL-6. As summarized in Figure 3(c), the CCK-8 assay showed that the rate of proliferation was higher in IL-6-treated MDSCs compared with the control cells (Figure 3(d)). CFSE further demonstrated that IL-6 promoted the proliferation of MDSCs (Figure 3(e)). The flow cytometry images also showed that IL-6 increased the proportion of MDSCs in both humans and mice (Figures 3(f) and 3(g)). These results showed that IL-6 accelerated MDSC accumulation by promoting proliferation.

### 3.4. IL-6 Improved the Immune-Suppressive Activity of MDSCs

**Figure 4:**
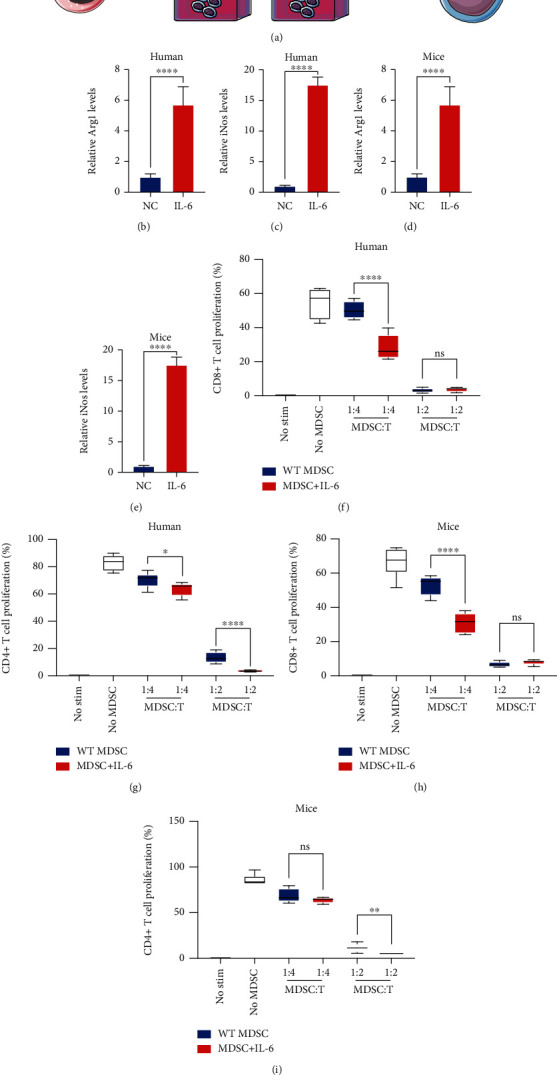
IL-6 improved the immune-suppressive effects of MDSCs. (a) Experimental diagram of MDSC extraction and IL-6 treatment. (b–e) RT-PCR was used to evaluate *Arg1* and *iNOS* levels in MDSCs with or without IL-6 treatment. (f–i) Summary of the suppressive effect of MDSCs by coculture with CD8+ T cells and CD4+ T cells at 1 : 4 and 1 : 2 ratios. Mean ± SD, ^∗^*P* < 0.05, ^∗∗^*P* < 0.01, ^∗∗∗^*P* < 0.005, and ^∗∗∗∗^*P* < 0.001. ns: no statistical significance.

MDSCs were sorted from human and mouse MIBC tissues to explore whether IL-6 influenced the immune-suppressive effects of MDSCs. MDSCs treated with or without IL-6 were collected and prepared for follow-up experiments (Figure 4(a)). RT-PCR showed that IL-6 treatment increased *Arg1* and *iNOS* levels (Figures 4(b)–4(e)). MDSCs were cocultured with CD8^+^ T cells or CD4^+^ T cells at different ratios (T cells : MDSCs = 1 : 4 or 1 : 2, respectively). IL-6-treated MDSCs significantly impeded the proliferation of CD8^+^ T cells and CD4^+^ T cells (Figures 4(f)–4(i)). Accordingly, IL-6 promoted the proliferation of MDSCs and immune-suppressive activity.

### 3.5. IL-6 Activated the MAPK Signaling Pathway in MDSCs

**Figure 5:**
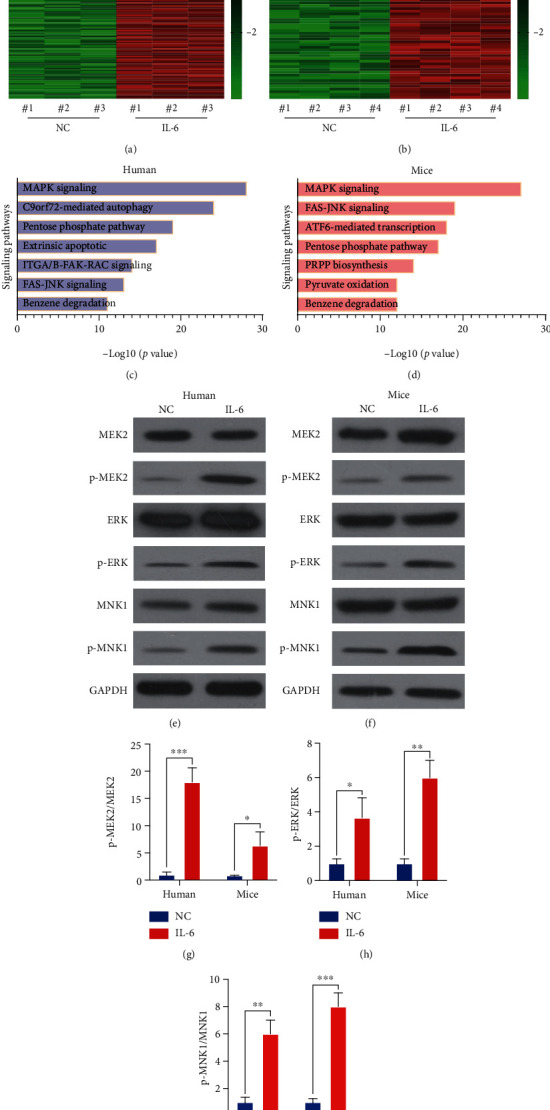
IL-6 activated the mitogen-activated protein kinase (MAPK) signaling pathway in MDSCs. (a, b) Heat map of RNA sequencing results to evaluate differentially expressed genes in MDSCs with or without IL-6 treatment. (c, d) Differentially expressed genes were enriched for several signaling pathways. (e, f) Phosphorylation of critical proteins in the MAPK signaling pathway (MEK2, ERK, and MNK1) was evaluated by western blotting. (g–i) Intensity ratio of each band. Mean ± SD, ^∗^*P* < 0.05, ^∗∗^*P* < 0.01, and ^∗∗∗^*P* < 0.005.

MDSCs were collected from human and mouse MIBC tissues to explore the specific mechanism underlying the effect of IL-6 on MDSCs. MDSCs were treated with IL-6 for 48 h and used for RNA sequencing (Figures 5(a) and 5(b)). Differentially expressed genes were enriched for several signaling pathways, especially the MAPK signaling pathway (Figures 5(c) and 5(d)). To confirm this result, WB analysis was performed to evaluate the key proteins with or without phosphorylation in the MAPK signaling pathway (MEK2, ERK, and MNK1). IL-6 treatment induced the phosphorylation of MEK2, ERK, and MNK1 in MDSCs from both human and mouse MIBC tissues (Figures 5(e)–5(i)). These results indicated that IL-6 promoted the proliferation and immune-suppressive effect of MDSCs by upregulating the MAPK signaling pathway.

### 3.6. IL-6 Promoted MDSC Proliferation and Immune-Suppressive Effects by Activating the MAPK Signaling Pathway

**Figure 6:**
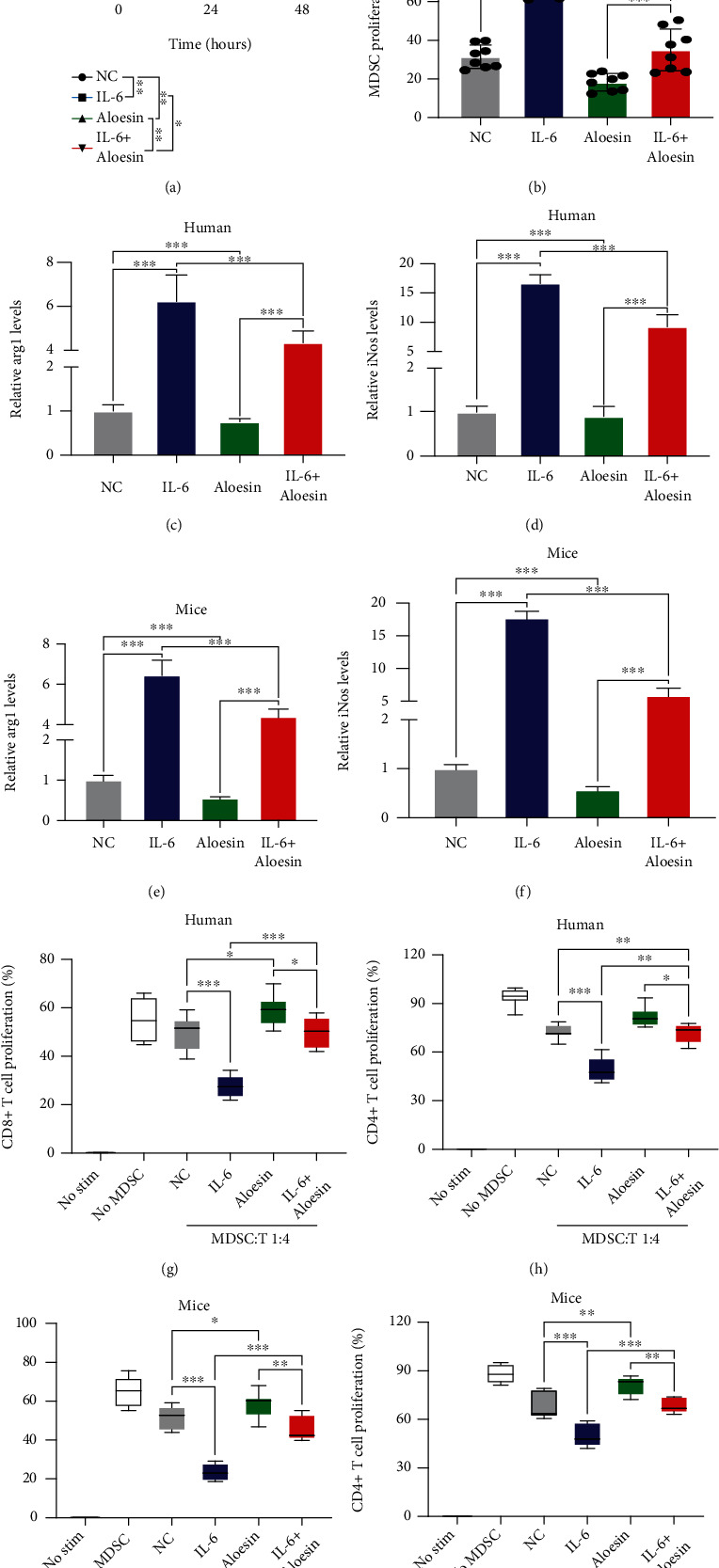
IL-6 promoted MDSC proliferation and immune-suppressive effects by activating the MAPK signaling pathway. (a) CCK-8 assays were used to evaluate MDSCs with or without IL-6 and/or aloesin treatment. (b) MDSC proliferation rates were evaluated according to fluorescence attenuation after IL-6 and/or aloesin treatment. (c–f) RT-PCR was used to evaluate *Arg1* and *iNOS* levels in MDSCs with or without IL-6 and/or aloesin treatment. (g–j) Summary of the suppressive effect of MDSCs by coculture with CD8^+^ T cells and CD4^+^ T cells at 1 : 4 ratios. Mean ± SD, ^∗^*P* < 0.05, ^∗∗^*P* < 0.01, and ^∗∗∗^*P* < 0.005.

**Figure 7:**
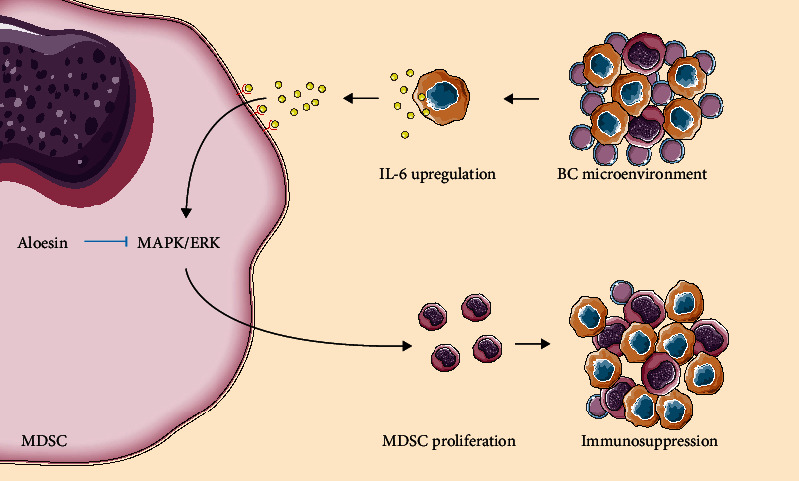
Schematic model. IL-6 promotes the proliferation and immunosuppressive function of MDSCs via the MAPK signaling pathway in MIBC.

Aloesin was used to block the activation of the MAPK signaling pathway so as to confirm the role of the MAPK subfamilies in MDSC proliferation and immune-suppressive effects. The CCK-8 assay and flow cytometry results showed that aloesin partially attenuated the IL-6-induced increase in proliferation (Figures 6(a) and 6(b)). RT-PCR showed that IL-6 treatment increased *Arg1* and *iNOS* levels, while IL-6 could reverse this phenomenon (Figures 6(c)–6(f)). MDSCs were cocultured with CD8^+^ T cells or CD4^+^ T cells at specific ratios (MDSCs : T cells = 1 : 4). IL-6-treated MDSCs significantly impeded the proliferation of CD8^+^ T cells and CD4^+^ T cells, which was partially reversed by aloesin (Figures 6(g)–6(j)). These results elucidated that IL-6 promoted the proliferation of MDSCs and immune-suppressive effects, and these effects could be reversed by targeting the MAPK signaling pathway. Taken together, IL-6 promoted the proliferation and immunosuppressive function of MDSCs via the MAPK signaling pathway in MIBC (Figure 7).

## 4. Discussion

IL-6 is highly pleiotropic and has a wide range of functions in various diseases [16]. The TME of most malignant lesions shows immune cell enrichment with immunosuppressive phenotypes, often via the immunomodulatory activities of IL-6-family cytokines. The members of this family have been identified as diagnostic or prognostic biomarkers of the response to therapy and disease activity in various cancers [17]. Aberrant IL-6 family overexpression and downstream receptor signaling pathway stimulation are frequent cytokine events in carcinomas and are often correlated with poor clinical outcomes [18]. The tumorigenesis-promoting effects of IL-6 cytokine family members are mediated via direct effects on proliferation, survival, migration, invasion, and metastasis in cancer cells and indirect effects on immunosuppression, modulation of inflammation, and angiogenesis in the interstitial stromal cells, which form the local TME [19]. IL-6 is expressed in numerous BC cell lines and is more abundant in the specimens of patients with MIBC than those with NMIBC [20]. Both BC cell lines and primary BC tissues express IL-6 and IL-6 receptor protein [21]. IL-6 clinically serves as an alternative marker of host immunity in patients with BC [22]. The canonical activation of immunity in BC elicits the production of IL-6, leading to BC-associated immune malfunction [23]. Typically, serum IL-6 level is significantly higher in higher-grade patients than in low-grade patients; furthermore, it is linked to patients with recurrent BC [24]. Patients with elevated IL-6 levels have a high risk of distant metastases and lymphatic metastases [25].

Among IL-6 cytokine family members, IL-6 has the most well-defined effect on the TME by prompting chronic inflammatory responses, regulating tumor vasculogenesis and the outgrowth of heterogeneous cells, and preventing Th1 cell-mediated antitumor immunocytotoxicity [26]. These protumorigenic effects are mediated by the recruitment, retention, activation, and function of TILs and reflect the various innate and adaptive immune responses regulated by IL-6 in immunity and chronic inflammatory diseases [27]. In melanoma, however, IL-6 exerts antitumor effects in the early stages of disease progression and later promotes tumor angiogenesis and the recruitment of immunosuppressive myeloid cells, such as MDSCs, to the TME [28]. Additionally, the tumor-promoting effects of IL-6 involve increases in MDSCs and Th17 cells, suppression of DCs and cytotoxic T cells, and phenotypic switching of tumor-associated macrophages (TAM) from an antitumor M1 phenotype to an immunosuppressive M2 phenotype [26]. In gastric cancer, serum-derived IL-6 activates and induces MDSCs, which express arginase 1, via the PI3K-Akt signaling pathway, thereby suppressing cytotoxic T cell functionality [29]. In hepatocellular cancer, the interaction between MDSCs and IL-6 promoted the generation of a chemoresistant phenotype [30]. However, in BC, low IL-6 levels could inhibit the activation of STAT3, promoting the formation of MDSCs [31]. The IL-6-induced experiments were simply performed on MDSCs in the serum of patients with BC. The serum MDSCs might not completely represent MDSCs in the TME of BC where elusive cell-cell communications are abundant, and MDSCs can be immunomodulated and undergo various alterations. The present analysis showed that IL-6 could stimulate the proliferation of MDSCs from the BC microenvironment in a time- and dose-dependent manner. Also, it indicated that further analysis, such as dose-dependent experiments for IL-6-induced MDSC alteration, should be implemented and more details should be elucidated.

MDSCs are primary inhibitors of efficient antitumor immunity as well as T cell proliferation and immune activity [32]. This study found that the proliferation of MDSCs correlated with increasing levels of IL-6; mice with high levels of IL-6 had increased frequencies of MDSCs. Patients with high frequencies of MDSCs had poorer overall survival compared with patients with low frequencies of these myeloid cells [33]. Peripheral MDSCs have a high correlation with tumor progression and grade, and low MDSC levels in circulation are significantly associated with an improved prognosis and outcomes in BC [34].

This study investigated the mechanisms by which IL-6 activated MDSCs, identifying the important role of the MAPK signaling pathway in MDSC proliferation in MIBC. Nevertheless, the precise interactions between IL-6 and MAPK are still unclear. Bongartz et al. reported that IL-6-induced MAPK activation could be categorized into a Gab1-independent early phase and a Gab1-dependent late phase. Gab1-independent MAPK activation in the early phase is vital for the follow-up launch of the Gab1-dependent expansion of MAPK activation, with the binding of the IL-6 receptor complex and SH2 domain-containing phosphatase 2 (SHP2). The successive synergetic recruitment of Grb2 and SHP2 to Gab1 is crucial for the Gab1-dependent augmentation of IL-6, eliciting MAPK signaling pathway activation in the late phase and the upregulation of various genes [35]. Additionally, MAPK cascade activation caused by IL-6 relies critically on the recruitment of SHP2 to Y759 of phosphorylation in the cytoplasmic territory of gp13063 [36].

ERK, a member of the MAPK family, is a survival-promoting factor in immunocytes, indicating that its activity may harbor immune-stimulating or immune-suppressive effects relying on the cell types penetrating the solid tumor [37]. ERK can activate the amplification of CD8^+^ TILs and promote the secretion of lytic granules and cytokines, upgrading the cytotoxicity of TILs in the TME [38]. The critical role of immature MDSCs in immune evasion was mediated by ERK in the present study. Generally, a subset of MDSCs shared numerous similarities with TAM; however, the upregulation of the MAPK pathway could polarize macrophages from the M2 phenotype to M1 phenotype and lead to the inhibition of the suppressive effects of MDSCs by the stimulation of LPS [39]. STAT3 inhibition could decrease MAPK and lead to the apoptosis of MDSCs [40]. In BC, tumor cells induced MDSC aggregation and expansion in the TME via the CXCL2/MIF-CXCR2 signaling pathway and the elevated phosphorylation of p38, ERK, and p65 [41].

In summary, the data suggested that IL-6 promoted MIBC progression by not only accelerating proliferation but also improving the immune suppression ability of MDSCs via activating the MAPK signaling pathway.

## Data Availability

The detailed procedures of methods and figures are attached. The data used to support the findings of this study are available from the corresponding author upon request.

## Abbreviations

BC: Bladder cancer
NMIBC: Non-muscle-invasive bladder cancer
MIBC: Muscle-invasive bladder cancer
TIL: Tumor-infiltrating lymphocyte
TME: Tumor microenvironment
MDSC: Myeloid-derived suppressor cell
IL-6: Interleukin 6
DC: Dendritic cell
MAPK: Mitogen-activated protein kinase
ERK: Extracellular signal-regulated kinase
ELISA: Enzyme-linked immunosorbent assay
CFSE: Carboxyfluorescein diacetate succinimidyl ester
RT-PCR: Reverse transcription-polymerase chain reaction
HPRT: Hypoxanthine phosphoribosyltransferase
SD: Standard deviation
CCK-8: Cell Counting Kit-8
ARG1: Arginase 1
iNOS: Inducible nitric oxide synthase
MEK2: Mitogen-activated protein kinase 2
MNK1: Mitogen-activated protein kinase-interacting kinase 1
PI3K: Phosphoinositide 3-kinase
AKT: Protein kinase B
Stat3: Signal transducer and activator of transcription 3
Gab1: GRB2-associated-binding protein 1
SHP2: SH2 domain-containing phosphatase 2
LPS: Lipopolysaccharide
CXCL2: C-X-C motif chemokine ligand 2
CXCR2: C-X-C chemokine receptor 2
AOD: Average optical density
MFI: Median fluorescence intensity.

## Additional Points

*Précis*. IL-6 strengthens the immunosuppressive ability of MDSCs via the MAPK signaling pathway in MIBC.

## Supplementary Materials

Supplementary Materials Table S1: monoclonal antibodies used for flow cytometric assay. Table S2: antibodies used for western blotting. Table S3: statistics of the RNA-seq data for human MDSCs. Table S4: statistics of the RNA-seq data for mice MDSCs.
