# Supplementary material for: IL-6 Promotes the Proliferation and Immunosuppressive Function of Myeloid-Derived Suppressor Cells via the MAPK Signaling Pathway in Bladder Cancer
Source: Biomed Res Int. 2021 Apr 23;2021:5535578. doi: 10.1155/2021/5535578 (PMC8088376; doi:10.1155/2021/5535578)
Supplement: Supplementary Materials — Table S1: monoclonal antibodies used for flow cytometric assay. Table S2: antibodies used for western blotting. Table S3: statistics of the RNA-seq data for human MDSCs. Table S4: statistics of the RNA-seq data for mice MDSCs. [file 5535578.f1.zip › table S1.docx]

| Cell Type | Antibody | Conjugate | Source | Product |
| --- | --- | --- | --- | --- |
| MDSCs (human) | CD45 | PE-cy7 | BioLegend | 368532 |
|  | CD11b | APC | BioLegend | 101212 |
|  | HLA-DR | PE-cy5.5 | Thermo Fisher Scientific | MHLDR18 |
|  | CD33 | PE | BioLegend | 303404 |
| MDSCs (mice) | CD11b | APC | BioLegend | 101212 |
|  | Gr-1 | PE-cy7 | BioLegend | 108416 |

Table S1. Monoclonal antibodies used for flow cytometric assay.
