# Supplementary material for: IL-6 Promotes the Proliferation and Immunosuppressive Function of Myeloid-Derived Suppressor Cells via the MAPK Signaling Pathway in Bladder Cancer
Source: Biomed Res Int. 2021 Apr 23;2021:5535578. doi: 10.1155/2021/5535578 (PMC8088376; doi:10.1155/2021/5535578)
Supplement: Supplementary Materials — Table S1: monoclonal antibodies used for flow cytometric assay. Table S2: antibodies used for western blotting. Table S3: statistics of the RNA-seq data for human MDSCs. Table S4: statistics of the RNA-seq data for mice MDSCs. [file 5535578.f1.zip › table S2.docx]

Table S2. Antibodies used for western blotting.

| Species Reactivity | Host species | Protein | Type | Source | Product |
| --- | --- | --- | --- | --- | --- |
| Human, Mouse | Rabbit | MEK2 | Monoclonal | Abcam | ab32081 |
|  |  | p-MEK2 | Polyclonal | Thermo Fisher Scientific | PA5-38140 |
|  |  | ERK1/2 | Monoclonal | Cell Signaling Technology | 4695S |
|  |  | p-ERK1/2 | Polyclonal | Cell Signaling Technology | 9101S |
|  |  | MNK1 | Monoclonal | Cell Signaling Technology | 2195S |
|  |  | p-MNK1 | Polyclonal | Cell Signaling Technology | 2111S |
|  |  | GAPDH | Monoclonal | Abcam | ab181602 |
| Rabbit | Goat | IgG | Polyclonal | Santa Cruz Biotechnology | sc-2004 |
