# Supplementary material for: IL-6 Promotes the Proliferation and Immunosuppressive Function of Myeloid-Derived Suppressor Cells via the MAPK Signaling Pathway in Bladder Cancer
Source: Biomed Res Int. 2021 Apr 23;2021:5535578. doi: 10.1155/2021/5535578 (PMC8088376; doi:10.1155/2021/5535578)
Supplement: Supplementary Materials — Table S1: monoclonal antibodies used for flow cytometric assay. Table S2: antibodies used for western blotting. Table S3: statistics of the RNA-seq data for human MDSCs. Table S4: statistics of the RNA-seq data for mice MDSCs. [file 5535578.f1.zip › table S3.docx]

| **Sample** | **A1** | **A2** | **A3** | **C1** | **C2** | **C3** |
| --- | --- | --- | --- | --- | --- | --- |
| before_filtering  _total_reads | 43530858 | 42112406 | 42276382 | 44760370 | 43434176 | 42917592 |
| before_filtering  _total_bases | 6529628700 | 6316860900 | 6341457300 | 6714055500 | 6515126400 | 6437638800 |
| before_filtering  _q20_bases | 6342839892 | 6139203789 | 6167303454 | 6525212568 | 6320126361 | 6243880091 |
| before_filtering  _q30_bases | 6057280736 | 5866525945 | 5892971644 | 6228800599 | 6014889253 | 5943202170 |
| before_filtering  _q20_rate | 0.971393656 | 0.971875728 | 0.972537252 | 0.971873493 | 0.970069646 | 0.969902209 |
| before_filtering  _q30_rate | 0.927660823 | 0.928709059 | 0.929277194 | 0.927725515 | 0.923219119 | 0.923195966 |
| before_filtering  _gc_content | 0.490095 | 0.489081 | 0.487808 | 0.487858 | 0.489655 | 0.501767 |
| after_filtering  _total_reads | 42939160 | 41548148 | 41748196 | 44157388 | 42777946 | 42271928 |
| after_filtering  _total_bases | 6357299212 | 6150456758 | 6185988078 | 6545683188 | 6353645100 | 6285495298 |
| after_filtering  _q20_bases | 6202836960 | 6004376062 | 6040656623 | 6388215051 | 6191545044 | 6124410877 |
| after_filtering  _q30_bases | 5939209068 | 5752477054 | 5786092269 | 6113429026 | 5908673883 | 5845536196 |
| after_filtering  _q20_rate | 0.975703165 | 0.976248805 | 0.976506347 | 0.975943208 | 0.974487077 | 0.97437204 |
| after_filtering  _q30_rate | 0.934234629 | 0.935292659 | 0.935354578 | 0.933963476 | 0.929965994 | 0.930004068 |
| after_filtering  _gc_content | 0.489863 | 0.488996 | 0.487649 | 0.487716 | 0.489509 | 0.50188 |
| ReadsFilter% | 98.64073894 | 98.66011455 | 98.75063576 | 98.65286636 | 98.48913906 | 98.49557263 |
| BaseFilter% | 97.36080724 | 97.36571464 | 97.5483676 | 97.49224128 | 97.52144026 | 97.63665675 |
| low_quality_reads | 590552 | 562902 | 525390 | 600096 | 651984 | 643120 |
| too_many_N_reads | 1146 | 1356 | 2796 | 2886 | 4246 | 2544 |
| too_short_reads | 0 | 0 | 0 | 0 | 0 | 0 |
| too_long_reads | 0 | 0 | 0 | 0 | 0 | 0 |

**Table S3** Statistics of the RNA-seq data for human MDSCs.
