# Supplementary material for: IL-6 Promotes the Proliferation and Immunosuppressive Function of Myeloid-Derived Suppressor Cells via the MAPK Signaling Pathway in Bladder Cancer
Source: Biomed Res Int. 2021 Apr 23;2021:5535578. doi: 10.1155/2021/5535578 (PMC8088376; doi:10.1155/2021/5535578)
Supplement: Supplementary Materials — Table S1: monoclonal antibodies used for flow cytometric assay. Table S2: antibodies used for western blotting. Table S3: statistics of the RNA-seq data for human MDSCs. Table S4: statistics of the RNA-seq data for mice MDSCs. [file 5535578.f1.zip › table S4.docx]

| **Sample** | **A1** | **A2** | **A3** | **A4** | **C1** | **C2** | **C3** | **C4** |
| --- | --- | --- | --- | --- | --- | --- | --- | --- |
| before_filtering  _total_reads | 45438521 | 43579769 | 45661477 | 49108733 | 45234756 | 44321789 | 47643125 | 48764315 |
| before_filtering  _total_bases | 6815779000 | 6536965100 | 6849221500 | 6941244400 | 6462351400 | 6825166600 | 6603467800 | 6875384300 |
| before_filtering  _q20_bases | 6671552664 | 6407158266 | 6687339801 | 6716306846 | 6364264813 | 6753124652 | 6468342356 | 6762135414 |
| before_filtering  _q30_bases | 6430186395 | 6187979187 | 6424226718 | 6543770304 | 6017564477 | 6424678201 | 6181518018 | 6537408963 |
| before_filtering  _q20_rate | 0.978831292 | 0.970456632 | 0.976423964 | 0.970895494 | 0.972134566 | 0.963135678 | 0.974562379 | 0.972323545 |
| before_filtering  _q30_rate | 0.949876617 | 0.944563467 | 0.931239925 | 0.953542388 | 0.963214778 | 0.943256489 | 0.935648623 | 0.946543218 |
| before_filtering  _gc_content | 0.493718 | 0.491187 | 0.494927 | 0.489433 | 0.492361 | 0.487651 | 0.487843 | 0.498016 |
| after_filtering  _total_reads | 45138199 | 43032147 | 43658442 | 46354897 | 42135432 | 43486531 | 46835314 | 44213548 |
| after_filtering  _total_bases | 6686543246 | 6451432461 | 6432186476 | 6456843214 | 6384331245 | 6687936315 | 6435897563 | 6532189654 |
| after_filtering  _q20_bases | 6432189756 | 6332148962 | 6326875213 | 6368732131 | 6246321563 | 6532168791 | 6362387534 | 6485963542 |
| after_filtering  _q30_bases | 6176541348 | 6035498322 | 6132486853 | 6285613214 | 6008348456 | 6335445321 | 6038735434 | 6238673512 |
| after_filtering  _q20_rate | 0.958643448 | 0.963218975 | 0.953548973 | 0.966873544 | 0.965321786 | 0.951324875 | 0.965431021 | 0.951010374 |
| after_filtering  _q30_rate | 0.938432178 | 0.930457852 | 0.920436584 | 0.946843102 | 0.954687351 | 0.937431214 | 0.924786351 | 0.938735232 |
| after_filtering  _gc_content | 0.492687 | 0.490564 | 0.491761 | 0.487432 | 0.491236 | 0.486832 | 0.487034 | 0.497853 |
| ReadsFilter% | 99.75643245 | 99.76543248 | 99.75613147 | 98.35431478 | 99.89351014 | 98.78943247 | 98.98731567 | 99.78942356 |
| BaseFilter% | 97.8974523 | 98.14564379 | 97.54867646 | 95.87513567 | 96.77984534 | 97.76434315 | 96.85321145 | 97.9865789 |
| low_quality_reads | 288643 | 252784 | 363227 | 340288 | 353110 | 248418 | 274632 | 237455 |
| too_many_N_reads | 5914 | 6913 | 6796 | 7431 | 5342 | 4886 | 5246 | 6544 |
| too_short_reads | 0 | 0 | 0 | 0 | 0 | 0 | 0 | 0 |
| too_long_reads | 0 | 0 | 0 | 0 | 0 | 0 | 0 | 0 |

**Table S4** Statistics of the RNA-seq data for mice MDSCs.
